# Quantitative Wood Anatomy—Practical Guidelines

**DOI:** 10.3389/fpls.2016.00781

**Published:** 2016-06-03

**Authors:** Georg von Arx, Alan Crivellaro, Angela L. Prendin, Katarina Čufar, Marco Carrer

**Affiliations:** ^1^Swiss Federal Institute for Forest, Snow and Landscape Research WSLBirmensdorf, Switzerland; ^2^Dipartimento Territorio e Sistemi Agro Forestali, Università degli Studi di PadovaPadua, Italy; ^3^Department of Wood Science and Technology, Biotechnical Faculty, University of LjubljanaLjubljana, Slovenia

**Keywords:** anatomical sample preparation, dendroanatomy, microscopic imaging, microtome sectioning, quantitative image analysis, QWA, tree-ring anatomy, wood sample collection

## Abstract

Quantitative wood anatomy analyzes the variability of xylem anatomical features in trees, shrubs, and herbaceous species to address research questions related to plant functioning, growth, and environment. Among the more frequently considered anatomical features are lumen dimensions and wall thickness of conducting cells, fibers, and several ray properties. The structural properties of each xylem anatomical feature are mostly fixed once they are formed, and define to a large extent its functionality, including transport and storage of water, nutrients, sugars, and hormones, and providing mechanical support. The anatomical features can often be localized within an annual growth ring, which allows to establish intra-annual past and present structure-function relationships and its sensitivity to environmental variability. However, there are many methodological challenges to handle when aiming at producing (large) data sets of xylem anatomical data. Here we describe the different steps from wood sample collection to xylem anatomical data, provide guidance and identify pitfalls, and present different image-analysis tools for the quantification of anatomical features, in particular conducting cells. We show that each data production step from sample collection in the field, microslide preparation in the lab, image capturing through an optical microscope and image analysis with specific tools can readily introduce measurement errors between 5 and 30% and more, whereby the magnitude usually increases the smaller the anatomical features. Such measurement errors—if not avoided or corrected—may make it impossible to extract meaningful xylem anatomical data in light of the rather small range of variability in many anatomical features as observed, for example, within time series of individual plants. Following a rigid protocol and quality control as proposed in this paper is thus mandatory to use quantitative data of xylem anatomical features as a powerful source for many research topics.

## Introduction

Quantitative wood anatomy as meant here investigates quantitatively how the variability in xylem anatomical features of trees, shrubs, and herbaceous species is related to plant functioning, growth, and environment, and often explores how these relationships change over time. Xylem performs a wide range of functions that are essential for plants to grow and survive. The xylem transports water, nutrients, sugars, and hormones; buffers water uptake and loss; supports the mass of the canopy plus loads from wind, snow, ice, fruits, and epiphytes; displays foliage and flowers to resources like light and pollinators. Many different ways have evolved to perform these functions, and as a consequence, there is an enormous diversity of xylem anatomies that can be spotted through a microscope. Moreover, wood anatomical features represent a natural archive for growth-environment relationships and plant functioning with intra-annual resolution (Fonti et al., 2010). In fact, xylem cells can be localized at a certain position within a specific annual growth ring (e.g., earlywood or latewood), which is linked to the time of their formation. The xylem anatomical structure is influenced during its development by internal and external factors (e.g., Fonti et al., 2010, 2013a; von Arx et al., 2012; Aloni, 2013; Carrer et al., 2015), and mal-adjusted xylem structure may even be responsible for tree mortality (e.g., Hereş et al., 2014; Pellizzari et al., 2016). Quantitative wood anatomy capitalizes on the xylem anatomical structures mostly fixed in the stems once the cells are mature, and often focuses on a small number of cell types such as conduits (vessels and tracheids), parenchyma (axial and radial), and fibers.

Xylem anatomical features in plants are numerous, and sometime concern very small and delicate details (IAWA Committee, 1989, 2004; Crivellaro and Schweingruber, 2015). This necessitates careful processing and high accuracy during quantification, but also analyzing a sufficiently large and representative subset of the wood sample (Arbellay et al., 2012; Scholz et al., 2013; Seo et al., 2014; von Arx et al., 2015a). In other words, quantitative wood anatomy requires high-quality, high-resolution, and often large images of properly collected and prepared anatomical samples. Improved sample preparation protocols for these needs have lately been developed (Gärtner and Schweingruber, 2013; Yeung et al., 2015). Furthermore, recent improvements in computer performance, automated image-analysis systems (von Arx and Dietz, 2005; Fonti et al., 2009; von Arx et al., 2013; von Arx and Carrer, 2014) and processing and interpretation of anatomical data (Carrer et al., 2015) nowadays allow to significantly increase the number of measured anatomical features. Together, these advancements are providing the basis to create unprecedented datasets in terms of size and quality, thus also allowing to use quantitative wood anatomy for an increasing number of different research topics such as climate-growth interactions (Olano et al., 2013; Castagneri et al., 2015; Rita et al., 2015), stress responses (Fonti et al., 2013b), tree functioning (Petit et al., 2011; Olson et al., 2014; Guet et al., 2015; Pfautsch et al., 2016), functional anatomical properties to identify tree provenances most resistant to climate change impacts (Eilmann et al., 2014), and wood formation (Cuny et al., 2014; Pacheco et al., 2015) and production (Cuny et al., 2015) processes. However, the production of data meeting high quality requirements necessitates following a strict multi-step procedure, to avoid artifacts and mistakes that can significantly influence the measurements. This is critical considering the relatively small range of variability of many anatomical features, in time series often between 5 and 20% from year to year (Fonti et al., 2007, 2015; Olano et al., 2013; von Arx et al., 2015a) as compared to even several fold in ring width.

This paper shows all sequential steps from sample collection to anatomical sample preparation and high-quality data production, and presents guidance and pitfalls of quantifying anatomical features. As such, it is intended to reflect the current state of the art for quantitative wood anatomy, particularly for the quantification of the most commonly investigated water-conducting xylem cells (conduits), but we anticipate that many aspects will be similar in other anatomical features of the xylem and even the phloem.

## From sample to anatomical data: guidance and pitfalls

### Step 1: collecting samples in the field

Quantitative wood anatomy aims to extract information from anatomical structures of stems, shoots, branches, roots, rhizomes, and even needles and leaf petioles of monocots and dicots. In many cases samples used for quantitative wood anatomy are taken with an increment borer. This tool was originally developed to collect samples for forest mensuration and dendrochronological investigations. When collecting increment cores for anatomical analyses, it is even more crucial than for other purposes to check the sharpness of the cutting edge of the borer's tip to avoid macro- and micro-cracks in the samples. This can be tested by punching out paper circles from a newspaper. Furthermore, it is very important to core in an exact radial direction, from the bark toward the pith, perpendicular to the axial direction of xylem cells, and keeping the borer in a fixed position while drilling. The use of a pusher is recommended when collecting cores for anatomical analyses. Cores of 10–12 mm in diameter are preferable compared to the standard 5 mm or smaller, to have more material to work with and to minimize the risk of fractures and twisting. Wood samples can also be extracted from stem discs obtained with a chainsaw, whereas in branches and smaller plant stems and/or root collars the entire samples can be processed. For the storage of wood samples we refer to literature such as Gärtner and Schweingruber (2013). Collection of herbs requires to excavate the root collar, e.g., with common garden tools. When cutting small branches, twigs, and small stems from a plant with pruners, the first (squeezed) part of the sample needs to be removed with a small-jagged saw (in hard samples) or a razor blade (in soft samples) before preparing microsections to avoid cracks and fragmentation.

### Step 2: preparing microsections

#### 2.1 General procedure

Typically, sample preparation involves producing microsections of 10–20 μm thickness with a sledge or rotary microtome, staining of the pallid cell walls with an agent as safranin, astrablue, toluidine blue, cresyl violet acetate, and their combinations to increase contrast in an anatomical slide (Gärtner and Schweingruber, 2013; Yeung et al., 2015). Boiling or just soaking the samples in water, embedding in paraffin, or using corn starch solution often helps to avoid damage to cell structures when cutting (Schneider and Gärtner, 2013; Yeung et al., 2015). For samples with very narrow cell lumina rice starch gives better results than corn starch because of the smaller grain size. When analyzing relatively large cells as the earlywood vessels in ring-porous species, it is usually sufficient and more efficient to smooth the wood surface by sanding or cutting (for instance with a core microtome, Gärtner and Nievergelt, 2010), removing sawdust and tyloses using high-pressure air or water blast, and increasing contrast of the wood surface with chalk powder and black marker (Fonti et al., 2009; Gärtner and Schweingruber, 2013).

#### 2.2 Microtome blades

**Figure 1:**
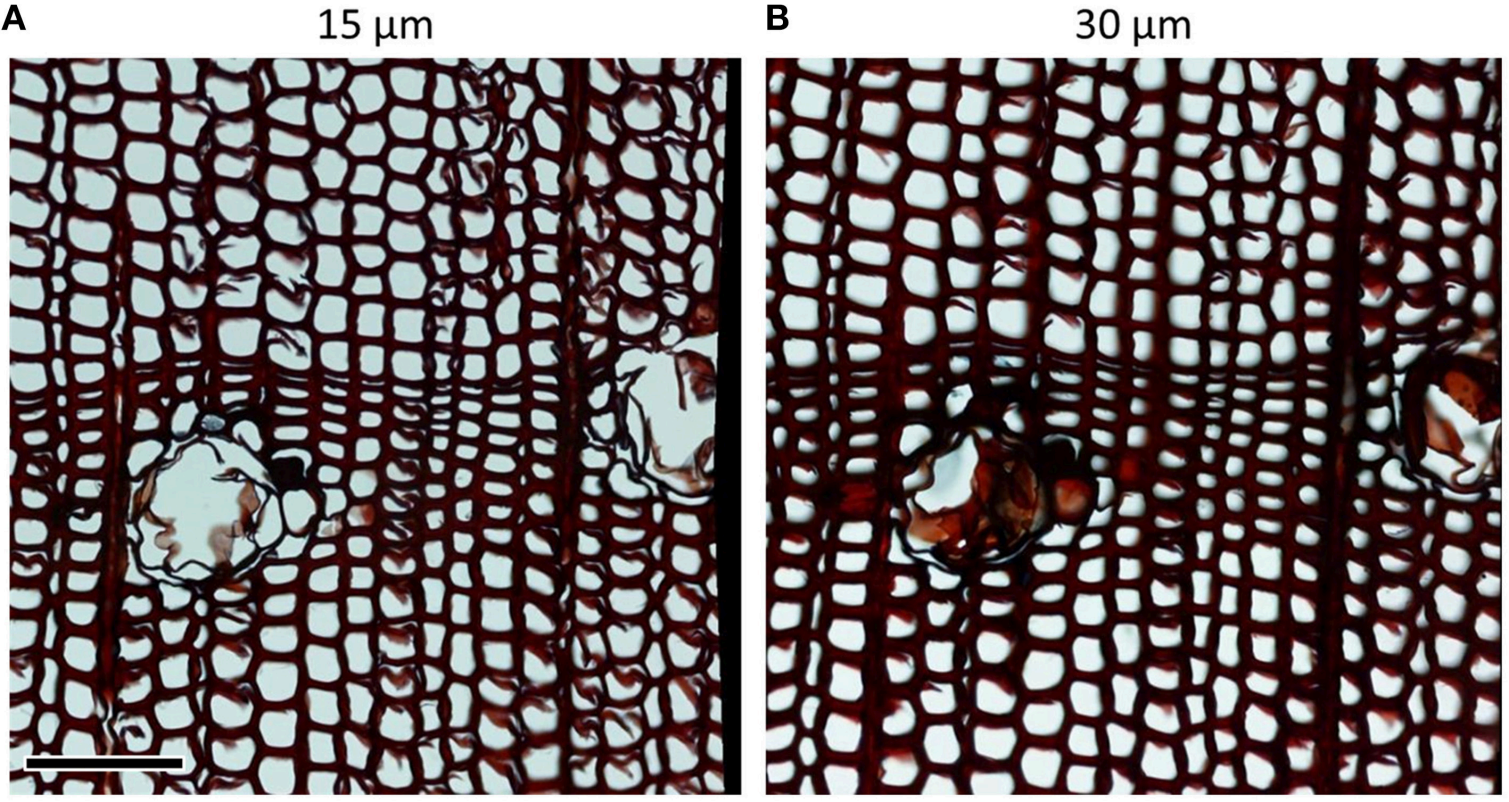
**Damage to cell walls due to dull blades in ***Pinus heldreichii*** cross-sections of (A) 15 μm and (B) 30 μm thickness**. In conifer samples, wall fragments rip off particularly easily at bordered pits. Such problems are aggravated in thinner sections as in panel **(A)**. Scale bar = 100 μm.

**Figure 2:**
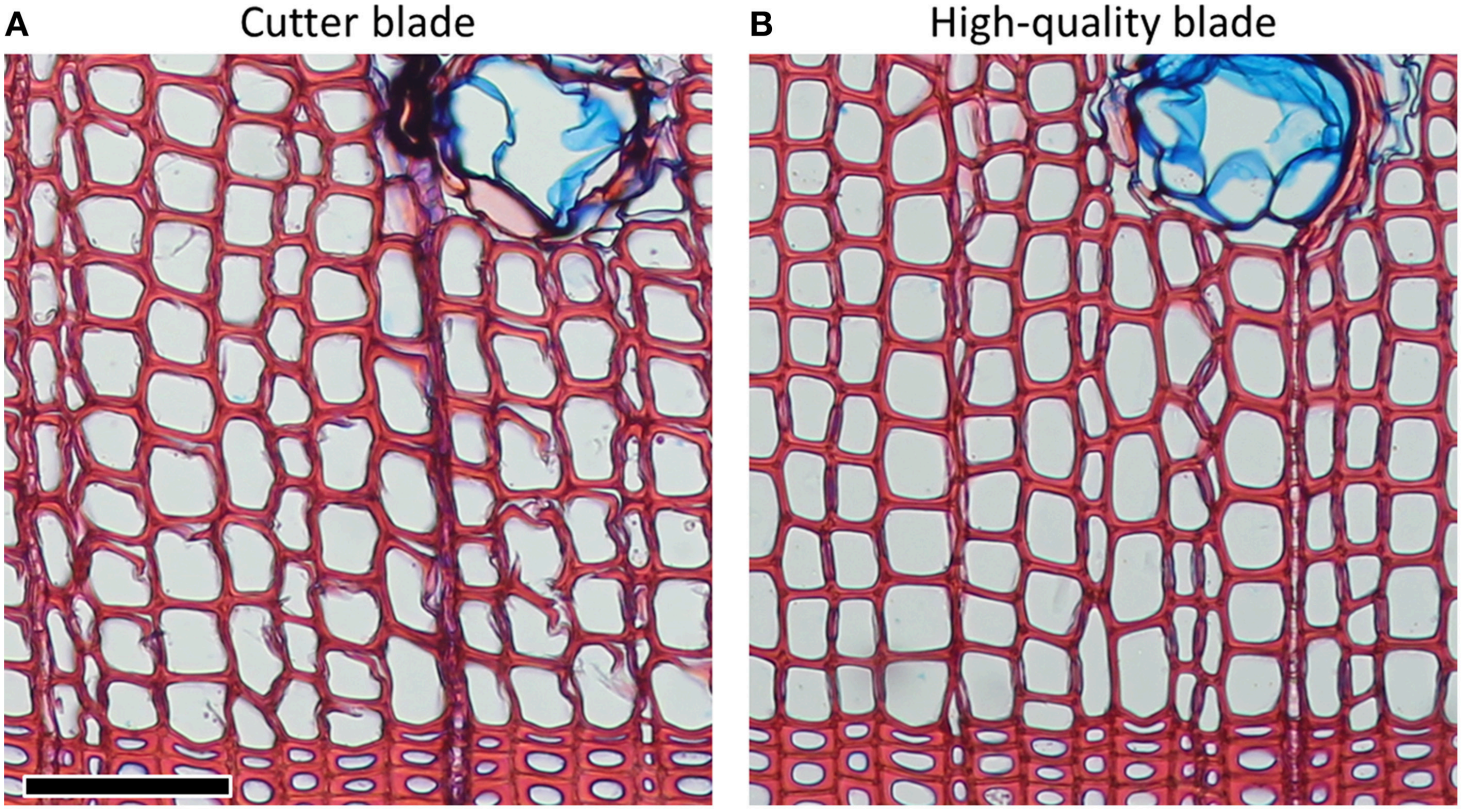
*****Pinus sylvestris*** cross-sections of 15 μm thicknesses from the same wood piece cut with (A) cutter and (B) high-quality blades**. Problems with disrupted cell structures can often be significantly reduced by using high-quality blades. Scale bar = 100 μm.

Microtome blades must be sharp and without defects to avoid disrupting the delicate anatomical structures. Damages due to dull blades are usually more pronounced in thinner sections (Figure 1). Frequent replacement or use of a previously unused part of the blade (often after cutting one sample, or after an even surface of the sample was prepared) can avoid this problem. Furthermore, using high-quality blades can significantly reduce cutting artifacts (Figure 2). For both conifer and angiosperm samples, good results were reported when using Leica DB80 LX and Leica 819 low-profile blades (Leica Biosystems, Wetzlar, Germany), and Feather N35HR and N35 blades (Feather Safety Razor Co., Ltd., Osaka, Japan; e.g., Prislan et al., 2013; Gričar et al., 2014; Pacheco et al., 2015; Pellizzari et al., 2016), however the optimal blade depends on the microtome model and the sample properties (e.g., density of the material, part(s) of the stem, moisture content) and therefore requires lab-specific testing. Generally, for cutting xylem, blade types designed for hard tissues should be used.

#### 2.3 Sample orientation while cutting sections

**Figure 3:**
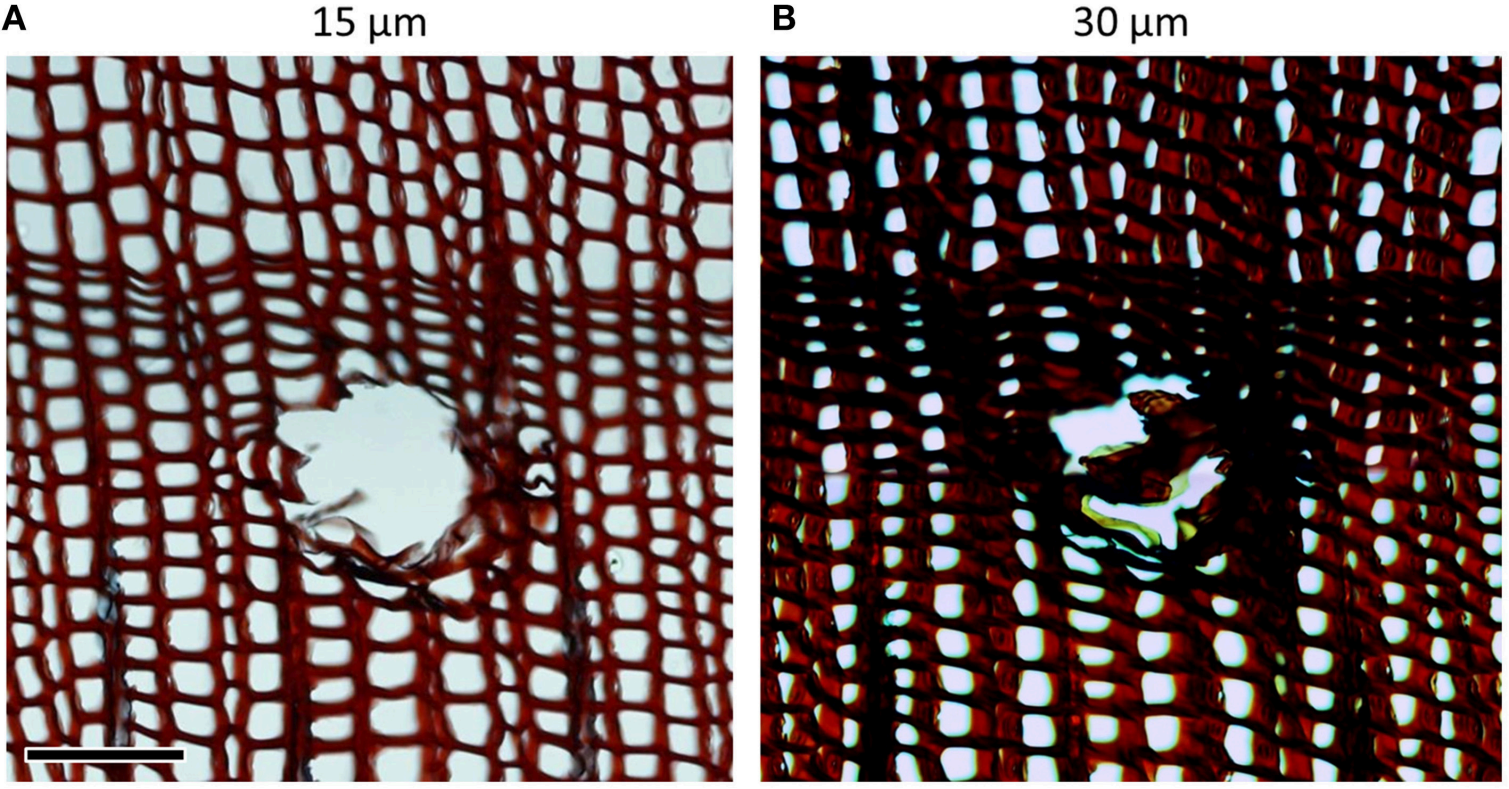
**Cross-sections of ***Pinus heldreichii*** cut from a not properly oriented sample, i.e., cutting direction that is not perpendicular to the axial tracheid orientation**. Non-orthogonal cross-sections result in underestimation of lumen area and overestimation of cell wall thickness. These measurement errors are weaker in **(A)** thinner than in **(B)** thicker sections as revealed after analyzing the entire images (c. 2500 cells; only subset images shown here) with the image-analysis tool ROXAS (cf. Table 1): mean cell lumen area in **(B)** was 43% smaller and mean tangential cell wall thickness 46% larger than in **(A)**. Scale bar = 100 μm.

When analyzing cross-sections, the wood samples should be cut perpendicular to the axially oriented xylem cells to avoid over- and underestimation of the measured anatomical features (Figure 3). When cutting longitudinal (i.e., radial and tangential) sections wood samples should be cut parallel to the axially oriented xylem cells. This is important when analyzing, for instance, rays in tangential sections. Measurement errors due to improper sample orientation increase with cutting thickness.

#### 2.4 Section thickness

**Figure 4:**
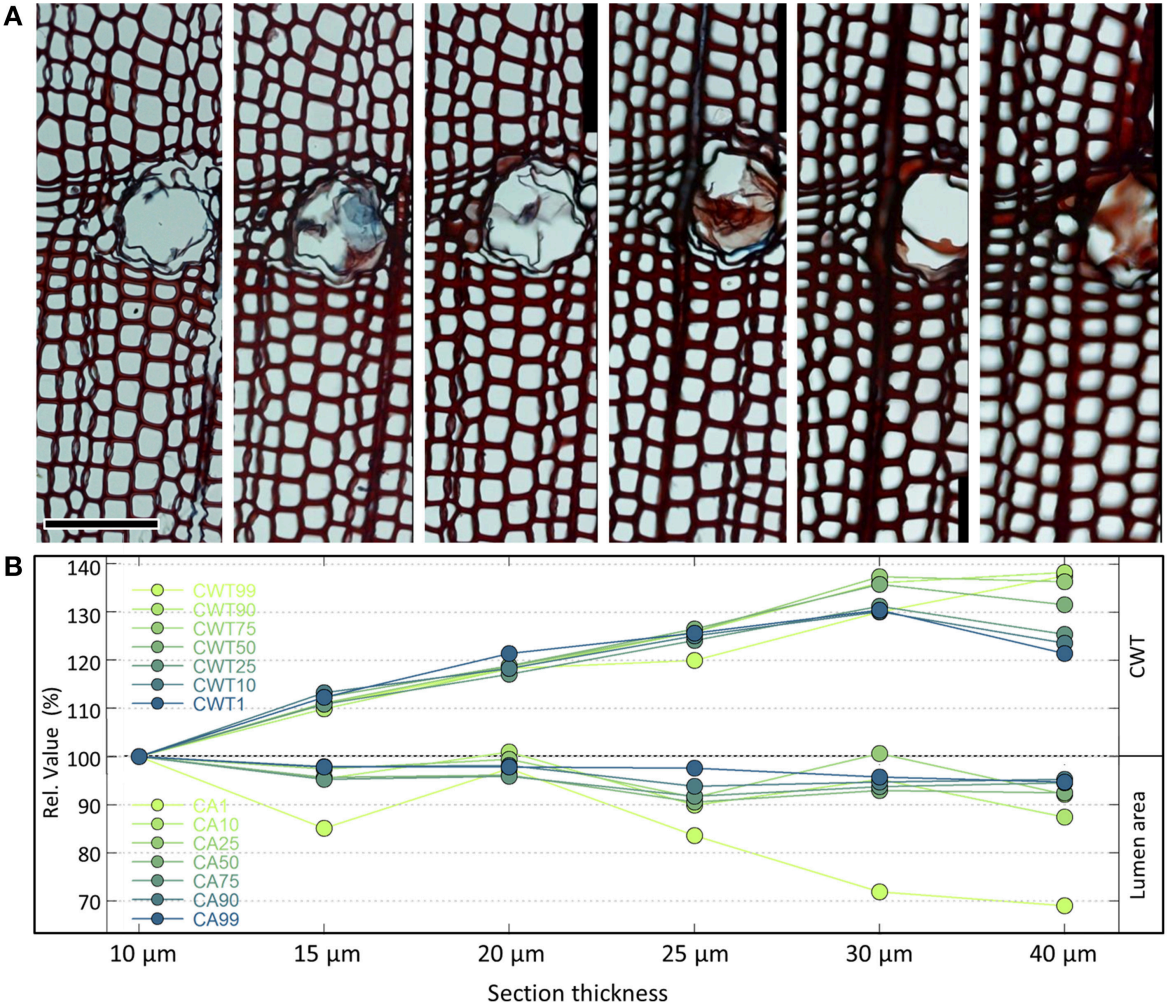
**(A)** Series of cross-sections of the same *Pinus heldreichii* wood piece using different cutting thicknesses from 10 to 40 μm (top row). The anatomical images are part of larger analyzed images containing each c. 4000 tracheids cells. The orientation of the samples is reasonably vertical, and images were produced keeping staining procedure and microscope settings standardized. Analyzing the images with the image-analysis tool ROXAS (cf. Table 1) using always the same settings reveals that the measured lumen area reduces markedly from the thinner to the thicker cross-sections **(B)**. This effect is stronger for smaller cells with a 31% reduction in the lowest percentile of the cell lumen population (CA1) than for the largest cells with only 4–6% reduction (CA90, CA99). In contrast, the mean tangential cell wall thickness appears also for the thinnest walls (CWT1, belonging to the largest cells) up to 30% larger in thicker compared to thinner cross-sections. For the thickest cell walls (CWT99, belonging to the smallest cells) the cutting-thickness error was up to 40%. Note that the quantification of the measurement errors is based on the shown example only. To a certain extent some of the cutting-thickness errors can be alleviated by adjusting the settings of the image analyses, particularly the segmentation threshold (see Section Image Segmentation and Figure 10). Scale bar = 100 μm.

A cutting thickness between 10 and 20 μm is usually optimal. Analyzing thick sections usually results in over- and underestimation of anatomical features such as cell wall thickness and cell lumen area (Figure 4). Thick sections also often appear out of focus. On the other hand, sections should not be too thin, since the tissue staining might be too weak to obtain target structures of sufficient contrast. Weak staining can be improved to a certain extent by prolonging the duration of the staining process or slightly increasing the concentration of the stain. In addition, sections from different species and even individuals can differ in staining intensity. However, as the example in Figure 4 shows, even in the optimal range the measured values can be influenced by different cutting thicknesses. It is therefore important to standardize cutting thickness for all samples of the same project. A good practice is also to record the thickness of each section, if not fully constant for all samples, thus allowing to relate any outliers to potential cutting-thickness effects during data analysis. It is also important to bear in mind that comparing absolute values among different projects could be biased if different cutting thicknesses were used.

#### 2.5 Making permanent slides

Permanent slide preparation is recommended to make specimens last over a long time. The procedure requires to dehydrate sections after staining, and a mounting medium (e.g., Canada balsam, Euparal, Eukitt) to permanently fix the sections between two glass slides (Gärtner and Schweingruber, 2013). To avoid buckling of the section, which impairs a uniform focus when capturing an image, the slide with the cover slip is sandwiched between PVC strips with a small magnet placed on the top of the slide on a metal plate to keep the sections flat and air bubbles out during drying. Canada balsam and Euparal require drying in the oven at 60°C for 12 h. Permanent slides, once prepared, can be used over and over again and can be stored for longer time periods than non-permanent slides.

### Step 3: microslide digitizing

#### 3.1 Cleaning slides and cover glasses

**Figure 5:**
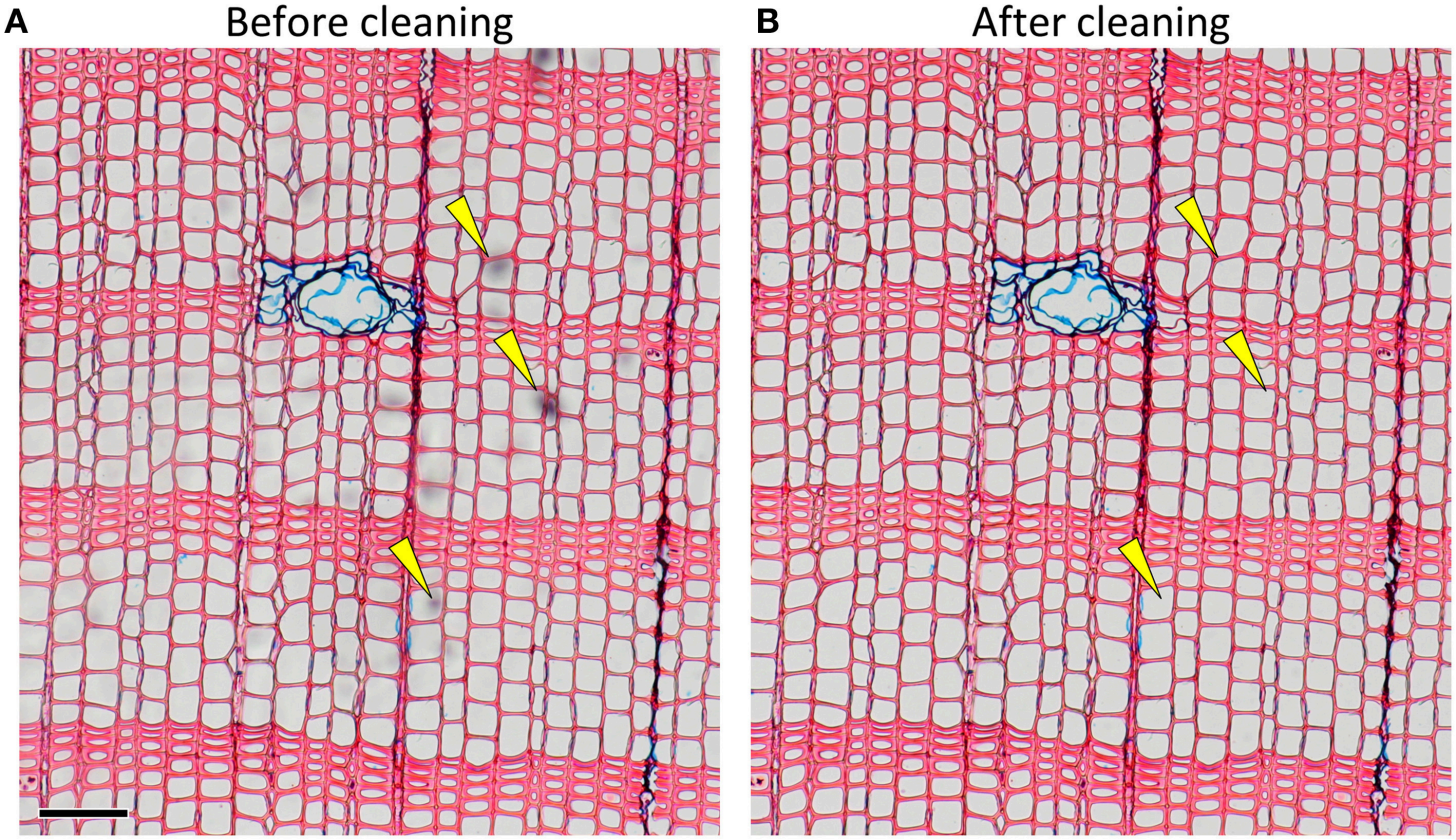
**Image of a slide with some pollution as indicated by yellow arrows (A) before and (B) after cleaning (***Pinus sylvestris***)**. Scale bar = 100 μm.

Pollution hampers automatic detection of anatomical features during image analysis and increases manual editing effort needed to obtain accurate data. Microslides should be cleaned carefully before capturing images to avoid obscured and low-contrast image parts (Figure 5). Frequent sources of pollution are, for instance excessive mounting medium (Gärtner and Schweingruber, 2013), fingerprints and dust particles. After drying, any hard mounting media on top of the cover slip can be scraped off with razor blades.

#### 3.2 Magnification

High-resolution digital images of anatomical sections are most commonly captured with a camera mounted on a optical microscope. Cameras integrated in the microscope system or standard cameras mounted with an appropriate adapter can be used. To observe and analyze conifers 10 × objectives are usually recommended, which, depending on the camera, can give a resolution of 1.7–2.5 pixels·μm^−1^. In angiosperms the 4 × objectives giving a resolution of 0.7–1.0 pixels·μm^−1^ are usually sufficient, especially for analyzing larger cells as vessels in trees, whereas smaller cells such as fibers also often require 10 × objectives.

#### 3.3 Contrast and illumination settings

**Figure 6:**
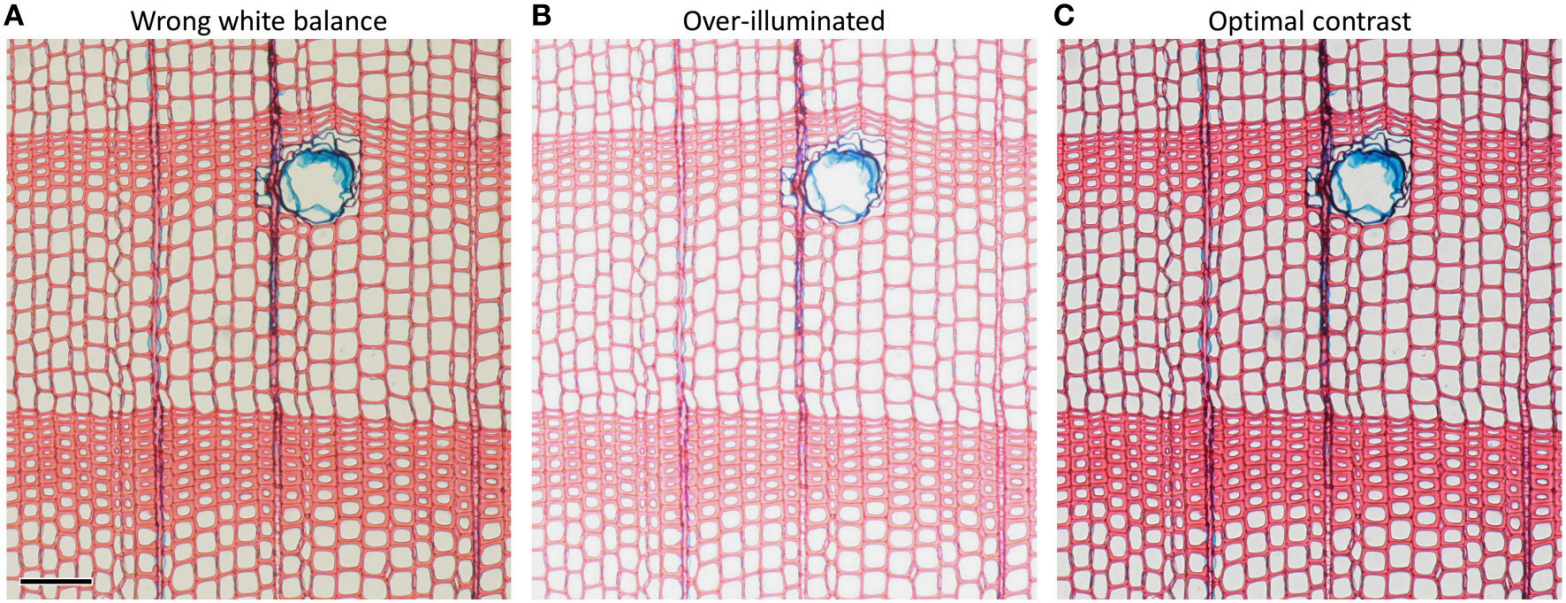
**Anatomical images of the same ***Pinus sylvestris*** microslide illustrating how imporper microscope settings such as (A) wrong white balance and (B) over-illumination reduce image contrast compared to (C) optimal settings**. Suboptimal microscope settings may impede automatic detection of anatomical features and result in under- and over-estimation of anatomical features. Scale bar = 100 μm.

Insufficient staining (due to too short staining time and/or old staining solutions) as well as wrong illumination, improper white balance and over-illumination lead to poor image contrast (Figure 6). Poor image contrast can significantly hamper the accurate automatic detection of anatomical structures during image analysis.

The quality and accuracy of the image critically depend on proper microscope settings. In this respect, the Köhler illumination method represents a major step to improve image quality (McCrone, 1980) and should be applied as a standard.

#### 3.4 Focusing

**Figure 7:**
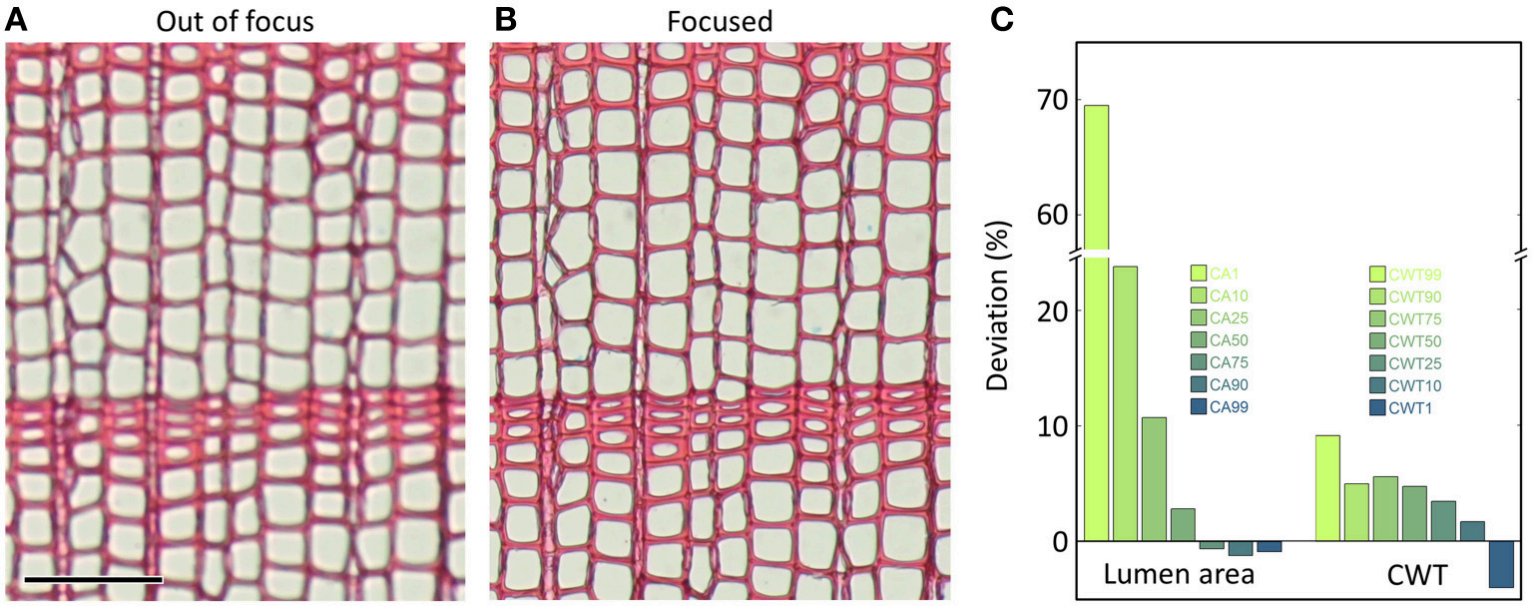
**The same anatomical microslide of ***Pinus sylvestris*** once captured (A) out of focus and (B) with optimal focus (only subset images shown)**. The entire images were analyzed with the image-analysis tool ROXAS (cf. Table 1) using always the same settings. In the out-of-focus image, 178 small tracheids out of totally 4240 (4.2%) were not detected, because lumina of very narrow tracheids were insufficiently defined. Accordingly, the lumen area corresponding to the smallest 1% of the measured values (CA1) were 69% larger in the poorly-focused than the well-focused image, while in the largest tracheids (CA99) the lumina appeared 1% smaller in the poorly-focused images **(C)**. Similarly, the thickest tangential cell walls (CWT99, corresponding to the very small tracheids) were overestimated by 9% in the poorly-focused compared to the well-focused image, while they were underestimated by 4% toward the thinnest walls (CWT1). Scale bar = 100 μm.

Careful focusing avoids obtaining blurred structures that can lead to measurement errors (Figure 7). Some systems offer automatic or semi-automatic focusing which contributes to consistently high image sharpness. When focusing manually, one should be aware that the live view on the computer screen is often of reduced size; therefore one should use a 100% zooming window for focusing, if available. When not all regions within an image frame can be in focus because of buckling, z- or focus stacking techniques, i.e., the combination of the focused image information from multiple images taken at different focal planes is a solution provided by some systems. Otherwise, the best and first solution would be to retry preparing a better microslide. In some wood samples this problem cannot be resolved even with careful microslide preparation. Then, excluding poorly focused regions from analysis is the best way to avoid measurement errors. Since the impact of poor focus depends on the size of the anatomical features (Figure 7), focusing the smaller target features (e.g., latewood lumina) is better than focusing larger target features (e.g., earlywood lumina).

#### 3.5 Scanning

For analyzing relatively large anatomical features such as the earlywood vessels in ring-porous species, it is possible to capture an image directly from the prepared wood surface with a flatbed scanner using an optical resolution of 1500–2500 dpi (Fonti et al., 2009). For permanent anatomical slides, slide scanners are an efficient alternative to optical microscopes, because they can produce high-resolution (e.g., 2.0 pixels·μm^−1^) images of entire anatomical samples, which avoids time-consuming image capturing and stitching (see next paragraph).

There are also several modifications of the aforementioned basic image capturing approaches, e.g., capturing images directly from the prepared wood surface with a dissecting microscope, thus combining efficient wood preparation with a higher optical resolution compared to flatbed scanners.

#### 3.6 Stitching composite images

**Figure 8:**
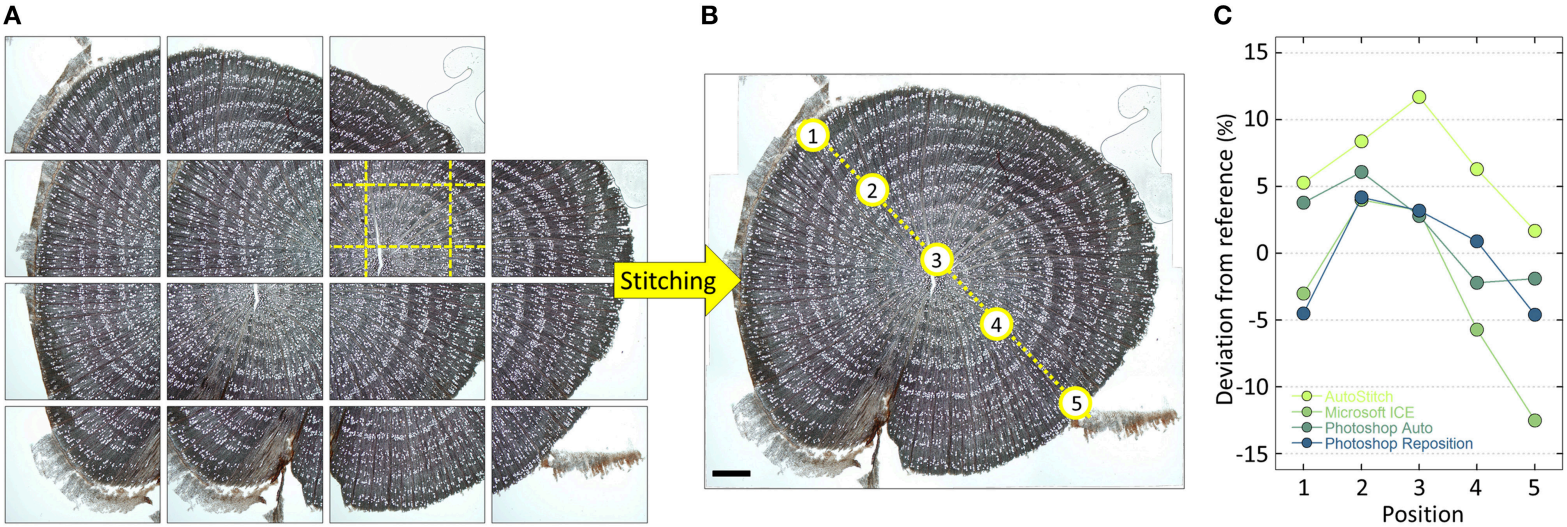
**(A)** Overlapping high-resolution images stitched together using PTGui and **(B)** the obtained high-resolution image of an entire *Verbascum thapsus* root cross-section. The used overlap with neighboring images is visualized for one of the images with yellow dashed lines in **(A)**. The input images contained distortions introduced by the used optical system, which were successfully removed by PTGui (verified by creating a composite image of a stage micrometer and measuring the distances between tick marks, which yielded constant values throughout the image). Five randomly selected vessels along a transect (see labels in **B**) having an lumen area between 100 and 3500 μm^2^ were subsequently measured using ROXAS (Table 1) using always the same settings in images stitched with the software PTGui, AutoStitch, Microsoft Image Composite Editor and Photoshop (Automatic and Reposition settings). Panel **(C)** shows the percentage deviation of the obtained values compared to the PTGui reference values. The values in all used stitching tools and settings deviate from the PTGui reference, thus indicating distortions. In addition, the magnitude of the deviations varied along the transect often changing from over- to under-estimation. Note that Photoshop Reposition setting also produces distortion-free images if input images are already distortion-free, while AutoStitch still introduces distortions. Scale bar = 1 mm.

Quantification of anatomical structures requires high-resolution images in order to obtain accurate data. However, higher magnification goes along with smaller field of view. This means that the anatomical sample often does not fit into a single image frame captured with an optical microscope, particularly when working with larger samples as the ones used, for example, to build time series of anatomical features (tree-ring anatomy or dendroanatomy). If no slide scanner is available (see above), this dilemma can be resolved by capturing several overlapping images and stitch them together (Figure 8).

For image stitching, overlapping images are produced using a microscope stage and systematically moving through the sample while capturing images. Re-focusing should be performed after every single or every few images. The overlap between individual images in angiosperm samples should be about 20% (Figure 8A), while in conifers we recommend about 30–40% to facilitate the stitching process. Overlapping images of a sample are then merged to an overall composite or panorama image using stitching software (Figure 8B). We recommend using specialized tools such as PTGui (New House Internet Services B.V., Rotterdam, NL) and AutoPano Pro (Kolor SAS, Francin, F) since they offer full control and reproducibility while producing distortion-free composite images. In contrast, some of the widely used stitching systems can produce distortions and artifacts which would lead to inaccurate results. With sufficient overlap and focused images PTGui and AutoPano Pro are usually able to create the composite image automatically. If not, both software allow to manually add control points, i.e., identical structures in the overlapping image parts. If the software are configured correctly, they are even able to correct any image distortions introduced by the optical system (Figure 8C; see von Arx et al., 2015b), e.g., when not using the recommended distortion-free “plan” type lenses.

### Step 4: quantifying anatomical features in anatomical images

#### 4.1 Image analysis tools

Once the image is produced, image-analysis tools are used to quantify the anatomical features. While target structures can be outlined and measured manually, automated image analysis allows to quantify a larger number of anatomical features in a much shorter time, and in an objective and reproducible way. Several image-analysis tools are used for quantitative wood anatomy. They differ considerably in functionality, ranging from rather general image analysis software such as ImageJ (Rasband, 1997–2016) to very specialized tools such as WinCELL (Regent Instruments Inc., Québec, Canada) and ROXAS (von Arx, www.wsl.ch/roxas; Table 1). The choice of the most appropriate tool depends on the specific needs. For a general characterization of xylem anatomical features in rather small samples a general tool is sufficient. However, if the sample depth in terms of number of trees, years, and anatomical features measured, but also the requirements in terms of specific and comprehensive output is important for the subsequent inferences, we recommend using specialized tools.

**Table 1:** **Overview of some image-analysis tools used for quantifying anatomical features**.

	**Tool**	**Cell size**	**Cell wall thickness**	**Tree-ring analyses**	**Cell filtering**	**Object model**	**Interactive editing**	**Some other features**	**License**
Specialized tools	ROXAS	Yes	Automatic	Automatic	Size, color, shape, context	Vector	Yes	- Analyzing large images (up to 1,000,000 cells) - Large set of anatomical output parameters - Automatic image processing - Batch processing options - Online library and customization of tailored configurations	Free, but requires commercial Image-Pro Plus (v6.1 or higher)
	WinCELL	Yes	Automatic	Manual	Size, color, shape	Pixel	Yes	- Large set of anatomical output parameters - Batch processing options - Customizing tailored configurations - Microscope and scanner control	Commercial
General tools	ImageJ	Yes	Manual	No	Size, shape	Vector	No	- Large collection of plugins by community - Macro development tools	Free
	CellProfiler	Yes	Manual	No	Size, color, shape	Vector	Yes	- Batch analyzing large sets (>1000) of images - Automating workflow using modules	Free
	Image-Pro Plus	Yes	Manual	No	Size, color, shape	Vector	Yes	- Powerful image processing and analysis functions - Macro development tools	Commercial
	NIS-Elements	Yes	Manual	No	Size, shape	Vector	Yes	- Macro development tools - Microscope control	Commercial
	AxioVision	Yes	Manual	No	Size, shape	Vector	Yes	- Automating workflow and macro development tools - Microscope control	Commercial

Despite the diversity of tools offering different levels of automation, specialization and usability, the way they are used to quantify anatomical features follows the same basic steps that are explained in the following.

#### 4.2 Determining the spatial image resolution

To obtain the measurements in metric units the pixel-to-micrometer resolution needs to be determined first. Some microscopic imaging systems provide this information directly, or add a spatial scale bar to the image that can be used as a reference. Where such information is missing, the best way to obtain the spatial resolution is to take a microscopic image of a stage micrometer or graticule (slide with an engraved high-accuracy micrometer scale) in the target magnification and measure several times the distance between two tick marks in pixels using a line tool. The obtained line length in pixels is then divided by the known line length in micrometers to receive the pixel-to-micrometer resolution. Selecting distant and different tick marks in each line measurement increases the robustness. In images from a flatbed scanner, the same information can be derived from the known resolution in dpi:

(1) $$\begin{array}{l} \frac{x}{25,400} \end{array}$$

Where *x* is the resolution of the scanned image in dpi. A resolution of 1500 dpi, for instance, corresponds to 0.059055 pixels·μm^−1^.

#### 4.3 Image processing

**Figure 9:**
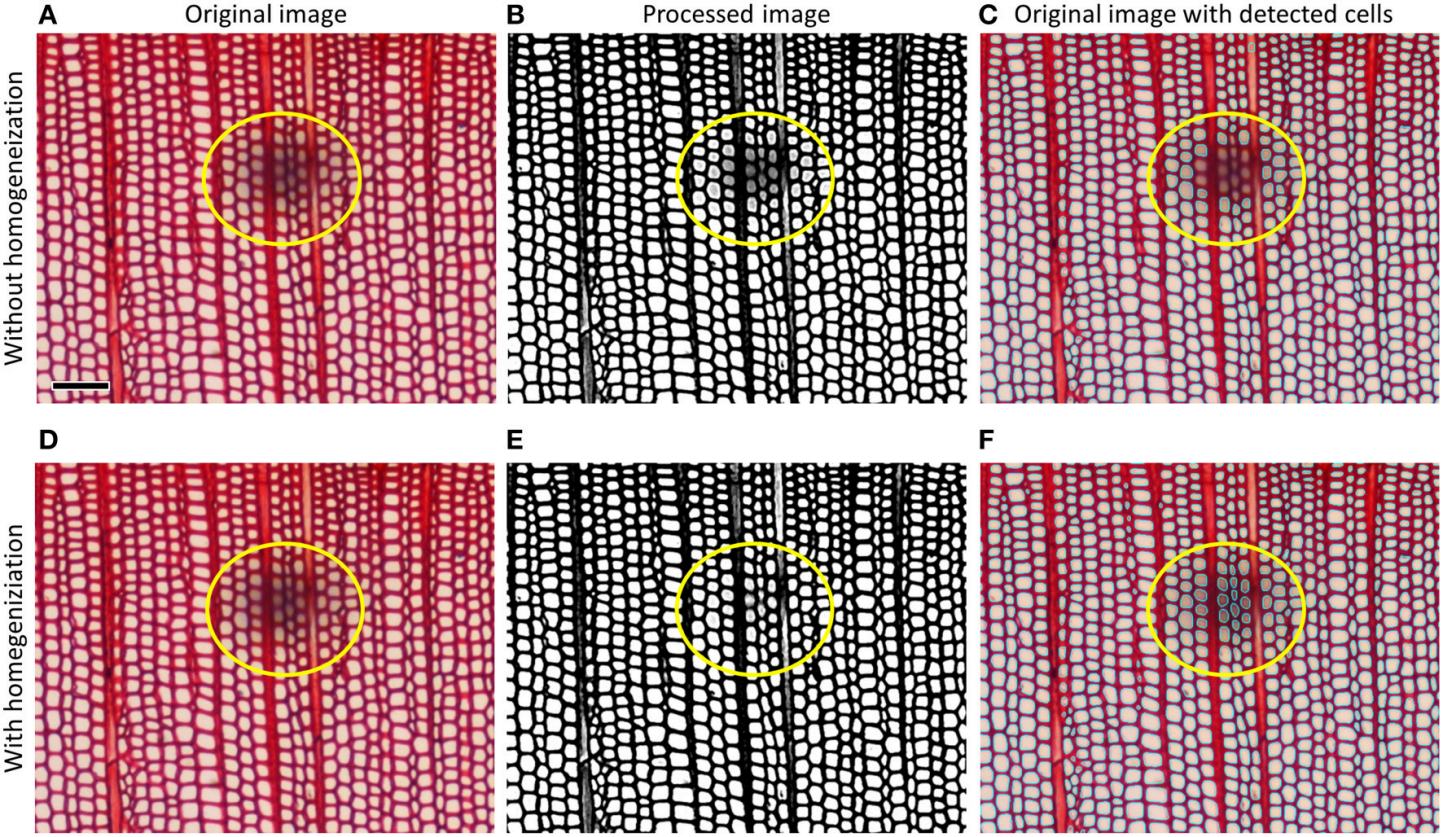
**Top row (A–C) shows how tracheid lumina obscured by a dust particle on the cover glass of a ***Pinus leucodermis*** sample remain undetected using ordinary image processing, bottom row (D–F) shows how contrast homogenization technique (using the image-analysis tool ROXAS in this case) allows to automatically detect all lumina**. Scale bar = 100 μm.

In images showing deficiencies, the next step is image processing, which helps to increase contrast and enhance edges of target anatomical structures. Some specialized image analysis tools do this automatically. The example in Figure 9 shows how an unremoved dust particle on a permanent slide (cf. Figure 5) is removed by contrast homogenization, thus resulting in a more complete recognition of tracheid lumina. In general, image processing should be used conservatively as it can change the dimensions of anatomical features in the image. Generally, the better the quality of the anatomical sample and image the less image processing is required to detect and quantify the targeted anatomical structures.

#### 4.4 Image segmentation

**Figure 10:**
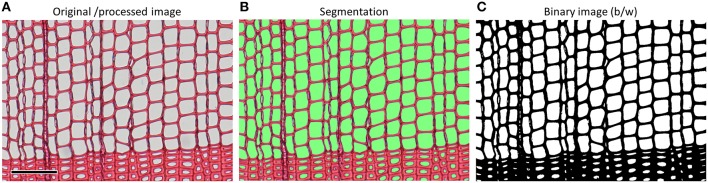
**(A)** Anatomical image of a *Pinus sylvestris* sample with **(B)** visualization of the segmentation threshold by a green mask and **(C)** the resulting binary image after performing the segmentation, which is the basis for quantifying the anatomical features. Depending on the image-analysis software the segmentation is applied to the original or processed color image, or a gray-scale image resulting from one to several image-processing steps (cf. Figure 9). Scale bar = 100 μm.

The original or processed image usually needs to be converted into a black-and-white (binary) image that allows discrimination between target and non-target structures (Figure 10). In this step called “segmentation” or “thresholding” a color or intensity value that optimizes this separation is—depending on the image-analysis tool—manually or automatically defined. Inhomogeneous image brightness and contrast due to inappropriate light source, uneven sample flatness or thickness and sample pollutions (cf. Figure 9) make it difficult or impossible to find a segmentation threshold that accurately discriminates between target and non-target structures in the entire image; such artifacts should therefore be avoided or corrected. The incorrect selection of a segmentation threshold can easily influence the data by 5–10%, particularly when the anatomical features in the image are not well defined because of poor contrast and focus. The segmented image is the basis for quantifying the anatomical features.

#### 4.5 Detecting and measuring anatomical features

**Figure 11:**
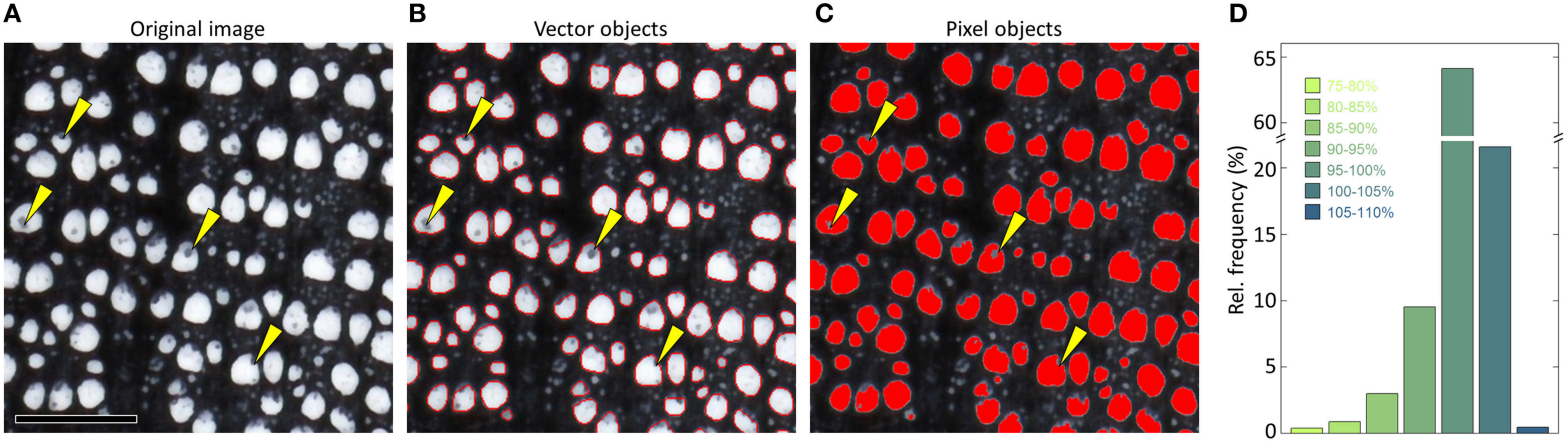
**Defining the anatomical features in a (A) sub-optimal image of ***Quercus petraea*** (surface scan, 2400 dpi) as (B) vector instead of (C) pixel objects allows to correct some sample artifacts, e.g., by applying a convex outline filter**. Panel **(D)** compares the percent deviation of vessel lumen area when representing the identical vessels in the selected image as pixels vs. vectors after analyzing the entire sample (>2500 vessels) with the image-analysis tool ROXAS. 20.2% of the measured values deviate by ≥5% from the supposedly more accurate vector object value, and 4.3% by ≥10%. While underestimation of lumen area in the pixel representation can be very strong due to artifacts as highlighted by the yellow arrows in **(A–C)**, pixel representation also resulted in slight overestimation (<5%) of 21.6% of all vessels because of pixel rounding effects. Note that some of these deviations can be significantly reduced by manual editing. Scale bar = 1 mm.

The segmented (binary) image is the basis for detecting and measuring anatomical features. Most image-analysis tools represent the anatomical features as vector instead of pixel objects (Table 1), which is usually better because irregularities can be corrected more easily (Figure 11), and the results are given in sub-pixel resolution.

#### 4.6 Improving score and accuracy of anatomical feature detection using filters

**Figure 12:**
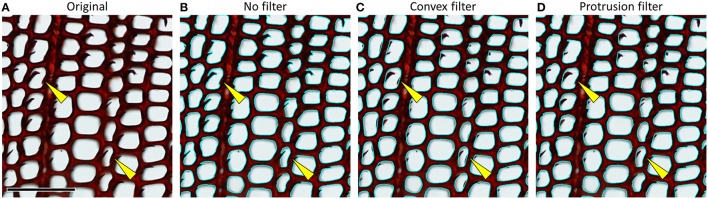
**(A)** Cross-section of a *Pinus sylvestris* wood piece showing ripped-off cell walls. **(B)** Same sample with overlay of detected lumen outlines (cyan) without any correction, resulting in measurement errors. **(C)** A convex outline filter can correct such artifacts, but may also cut off true concavities in the lumen outlines, e.g., due to pit inflections (see examples highlighted by yellow arrows), while **(D)** a more powerful “protrusion filter” (as implemented in the image-analysis tool ROXAS) better discriminates between artifacts and true concavities. Scale bar = 100 μm.

Most image-analysis tools include size filters to automatically exclude objects that are too small or too large. Moreover, specialized tools offer automatic filters based on color and shape (Table 1). Some specialized tools such as ROXAS also include shape corrections, e.g., to correct for particles and ripped-off cell walls that protrude into the cell lumen (Figure 12), and context-based filters that allow, for example, to filter out cells that strongly deviate from the closest neighboring cells.

#### 4.7 Manual editing

To obtain quality results and deal with image deficiencies, final manual editing is often necessary after automated detection and filtering of anatomical features. Specialized image-analysis tools offer efficient editing options for deleting, adjusting and adding anatomical features. However,—and this is a pivotal information—it is generally several times more efficient to invest time into high-quality anatomical slides and images rather than to manually improve a suboptimal automated feature detection.

#### 4.8 Xylem anatomical metrics and data storing

Specialized image-analysis tools automatically extract many metrics from the visual output and save them into data files, others offer manual export functions. Examples of primary, but also several derived anatomical metrics that are used to address many distinct research questions can be seen in the instruction film by von Arx et al. (2015b).

Among the primary measurements are:

- Width and calendar year of annual rings.
- Number, position and dimensions of conduits, resin ducts and rays (globally/within annual rings).
- Cell wall thickness (conduits, fibers).

Among the many derived metrics calculated manually or automatically by some image analysis tools are:

- Mean hydraulic diameter Dh (lumen diameter corresponding to the mean hydraulic conductivity of all conduits; Sperry et al., 1994).
- Conduit and resin duct density (no./mm^2^; Scholz et al., 2013).
- Vessel grouping indices (connectivity among vessels; von Arx et al., 2013).
- Mork's index (an indicator for anatomical wood density in conifers; Denne, 1989).
- Bending resistance index (t/b)^2^ (cell implosion safety; Hacke et al., 2001).
- Theoretical hydraulic conductance based on Poiseuille's law (Tyree and Zimmermann, 2002).

#### 4.9 Quality control

How much manual editing is needed? We recommend to define this by comparing the output of the target anatomical parameters after no, moderate and perfect manual editing for one to a few representative subset images (e.g., including 1000–2000 cells from both early- and late-wood). If all previous steps were done properly the output with no or moderate editing will not deviate from the (near to perfect) output obtained after heavy-editing by more than 1–2%; this is an accuracy we deem sufficient for most purposes.

## Conclusions

In this paper we provided some practical guidance and identified several pitfalls to successfully use quantitative wood anatomy in research. Producing xylem anatomical data is a challenging multi-step approach from sample collection to image analysis. As we showed with a few examples, potential measurement errors in many steps are between 5 and 20 or even 30%, which is in the same range as the variability of the anatomical metrics of interest, at least when excluding partly much stronger interspecific and ontogenetic variability. This is exacerbated by the fact that deficiencies in one step propagate to the next step, sometimes scaling up. The neglect of following a rigid and standardized procedure in terms of cutting thickness, staining, and illumination settings can therefore introduce considerable measurement errors and reduce the quality of the xylem anatomical dataset. While the specific measurement errors due to sample and image deficiencies can differ significantly within the smallest and the largest anatomical features, sometimes even changing from over- to underestimation, they are usually strongest in the smaller features such as latewood cell lumina and cell wall thickness. This is of particular relevance if the research goals are oriented towards, for example, intra-annual density profiles including maximum latewood density, or mechanical strength of cells. Although during image analysis the presented measurement errors can be reduced by defining specific settings for each image and manual editing, this is subjective, often very time-consuming, and generally still produces less accurate data than minimizing problems beforehand. The importance of producing high-quality anatomical slides and images can therefore not be stressed too much in terms of efficiency and accuracy. Then, quantitative wood anatomy is a very powerful tool that can give novel and mechanistic insights into the relationships between tree growth and environment over decades and even centuries.

## Author contributions

All authors planned and designed the research. GvA and Mc prepared the anatomical images. GvA performed the quantitative analyses. GvA wrote the first draft of the manuscript, which was finalized with contributions from all authors.

### Conflict of interest statement

The authors declare that the research was conducted in the absence of any commercial or financial relationships that could be construed as a potential conflict of interest.
